# COVID-19 Pandemic and Racial and Ethnic Disparities in Long-Term Nursing Home Stay or Death Following Hospital Discharge

**DOI:** 10.1001/jamanetworkopen.2024.56816

**Published:** 2025-01-24

**Authors:** Laurent G. Glance, Karen E. Joynt Maddox, Patricia W. Stone, Jingjing Shang, E. Yoko Furuya, Ashley M. Chastain, Ji Won Lee, Bridget Morse-Karzen, Andrew W. Dick

**Affiliations:** 1 Department of Anesthesiology and Perioperative Medicine, University of Rochester School of Medicine, Rochester, New York; 2 Department of Public Health Sciences, University of Rochester School of Medicine, Rochester, New York; 3 RAND Health, RAND, Boston, Massachusetts; 4 Department of Medicine, Washington University in St Louis, St Louis, Missouri; 5 Center for Advancing Health Services, Policy & Economics Research, Washington University in St Louis, St Louis, Missouri; 6 Columbia School of Nursing, Center for Health Policy, New York, New York; 7 Department of Medicine, Division of Infectious Diseases Columbia University Irving Medical Center, New York, New York

## Abstract

**IMPORTANCE:**

Long-term nursing home stay or death (long-term NH stay or death), defined as new long-term residence in a nursing home or death following hospital discharge, is an important patient-centered outcome.

**OBJECTIVE:**

To examine whether the COVID-19 pandemic was associated with changes in long-term NH stay or death among older adults with sepsis, and whether these changes were greater in individuals from racial and ethnic minoritized groups.

**DESIGN, SETTING, AND PARTICIPANTS:**

This cross-sectional study used patient-level data from the Medicare Provider Analysis and Review File, the Master Beneficiary Summary File, and the Minimum Data Set. Community-dwelling individuals aged at least 65 years hospitalized with sepsis between January 2016 and June 2021 were included. Data were analyzed from May to November 2024.

**EXPOSURE:**

Race and ethnicity and the COVID-19 pandemic.

**MAIN OUTCOMES AND MEASURES:**

Patients discharged alive experienced long-term NH stay or death if they resided in a nursing home more than 100 days after hospital discharge and had no period at home greater than 30 days, or died more than 30 days following hospital discharge. Interrupted time series analysis was used to evaluate the association between long-term NH stay or death and the pandemic and race and ethnicity.

**RESULTS:**

A total of 2 964 517 hospitalizations for sepsis of community-dwelling patients discharged alive (1 468 754 [49.5%] female; 19 549 [0.7%] American Indian or Alaska Native, 95 308 [3.2%] Asian or Pacific Islander, 282 646 [9.5%] Black, 279 011 [9.4%] Hispanic, 2 288 003 [71.2%] White individuals; mean [SD] age, 76 [8.3] years) were included. Compared with non-Hispanic White individuals, Black individuals were more likely to experience long-term NH stay or death (adjusted odds ratio [aOR], 1.33; 95% CI, 1.30-1.37; *P* < .001), while Asian or Pacific Islander (aOR, 0.79; 95% CI, 0.75-0.83; *P* < .001), Hispanic (aOR, 0.72; 95% CI, 0.70-0.74; *P* < .001), and American Indian or Alaska Native (aOR, 0.79; 95% CI, 0.72-0.87; *P* < .001) individuals were less likely to experience long-term NH stay or death. Long-term NH stay or death declined from 13.5% in the first quarter of 2016 to 6.9% in the first quarter of 2020. After adjustment, long-term NH stay or death decreased each quarter (aOR, 0.958; 95% CI, 0.957-0.959; *P* < .001) before the pandemic. The pandemic was associated with increased risk of long-term NH stay or death over time (aOR, 1.03; 95% CI, 1.02-1.04; *P* < .001 [each quarter]) compared with before the pandemic for non-Hispanic White individuals. The pandemic was not associated with differential changes in long-term NH stay or death for minoritized individuals compared with non-Hispanic White individuals.

**CONCLUSIONS AND RELEVANCE:**

In this cross-sectional study, older adults hospitalized with sepsis experienced an approximately 50% reduction in long-term NH stay or death over a 5-year period before the pandemic. These results suggest that during the pandemic, all individuals, regardless of race and ethnicity, experienced increased long-term NH stay or death compared with before the pandemic.

## Key Points

**Question:**

What is the association between the COVID-19 pandemic and long-term nursing home stay or death following hospital discharge among older community-dwelling adults discharged alive after a hospitalization with sepsis, and were these changes greater in individuals from racial and ethnic minoritized groups?

**Findings:**

This cross-sectional study of 2 964 517 hospitalizations for sepsis found that before the pandemic, Black individuals were more likely and other minoritized individuals were less likely to experience long-term nursing home stay or death compared with White individuals. White individuals and minoritized individuals experienced similar increases in long-term nursing home stay or death during the pandemic compared with before the pandemic.

**Meaning:**

These findings suggest that although the pandemic was associated with increased long-term nursing home stay or death, these increases were similar across all racial and ethnic groups.

## Teaser

This cross-sectional study investigates whether the COVID-19 pandemic was associated with changes in long-term nursing home stay or death following hospital discharge among older adults with sepsis, and whether these changes were greater in individuals from racial and ethnic minoritized groups.

## Introduction

The United States is in the midst of a demographic transition. Due to the aging of the Baby Boom generation and improvements in life expectancy, the number of people 65 years and older is expected to increase by 40% between 2022 and 2050 (from 57.8 million to 82.1 million) while the number of people 85 years and older is expected to increase by 170% (from 6.5 million to 17.4 million).^1^ These shifts in age distribution will be accompanied by shifts in the racial and ethnic makeup of older adults as an increasingly diverse population ages into older adulthood. With the aging of the US population, there is broad concern that our long-term care infrastructure will be inadequate in the coming years.^2^ Issues related to inconsistent and sometimes substandard care, insufficient staffing, high staff turnover, and the lack of patient-directed care all contribute to concerns about reliance on this sector for a growing elderly population.^3^ Furthermore, long-term care services are provided within the framework of a racially and socioeconomically segregated care system.^4^

Nursing home residents were disproportionately affected by the COVID-19 pandemic. Although nursing home residents represent 2% of Medicare beneficiaries, they account for 22% of COVID-19 cases.^5^ Moreover, nursing homes with the highest proportion of Black residents experienced a 3-fold higher number of COVID-19–associated deaths compared with those with the lowest proportion of Black residents.^6^ The pandemic’s disproportionate impact on nursing home patients and individuals from racial and ethnic minoritized groups within nursing homes has focused attention on long-term care in the US and has led to calls for transformative changes to address systemic racism in long-term care.^4^

In this context, there is a pressing need to understand the effect of the pandemic on patient-centered outcomes in the aging population. One such outcome is long-term nursing home stay or death (long-term NH stay or death), defined as new long-term nursing home residence following hospitalization or postdischarge death among adults who were community-dwelling before hospitalization. Although previous investigators have focused on the decline in functional status and discharge disposition immediately following hospitalization,^7,8^ focusing on new long-term nursing home residence yields a clearer picture of the true consequences of a hospitalization. Although the pandemic’s effect on postacute care has been previously evaluated,^9,10^ its association with long-term NH stay or death has not been reported. Our goal was to study the association between the pandemic and long-term NH stay or death among community-dwelling individuals aged 65 years and older hospitalized with sepsis, and whether individuals from racial and ethnic minoritized groups experienced a greater long-term NH stay or death than White individuals during the pandemic. We selected sepsis because it is one of the leading causes of death in the US, leads to high levels of postacute care, is associated with permanent reductions in cognitive function, and disproportionately affects minoritized individuals.^11,12,13^ This study aims to further our understanding of a key patient-centered outcome in older individuals hospitalized with a high-acuity illness overall, and in minoritized individuals in particular.

## Methods

This cross-sectional study was approved by the Columbia University institutional review board, which determined that informed consent was not necessary because it was exempt research on preexisting data. The Strengthening the Reporting of Observational Studies in Epidemiology (STROBE) reporting guideline was used to guide the reporting of this study.^14^

### Data Source

This retrospective cross-sectional study of community-dwelling adults discharged alive following hospitalization for sepsis was conducted using patient-level data from the 100% Medicare Provider Analysis and Review (MEDPAR) File, the Master Beneficiary Summary (MBS) file, and the Minimum Data Set (MDS) between 2016 and 2021. The MEDPAR and MBSF files includes information on demographic measures (age, sex, self-reported race, and ethnicity [American Indian, hereafter referred to as American Indian or Alaska Native; Asian or Pacific Islander; Black; Hispanic; non-Hispanic White, hereafter referred to as White; and other], which is captured at the time of Social Security enrollment and is coded using the Research Triangle Institute [RTI] Race Code^15^); *International Statistical Classification of Diseases and Related Health Problems, Tenth Revision (ICD-10)* diagnosis and procedure codes; date of admission; discharge destination, date of death; and hospital identifiers. The MDS contains assessment data on nursing home residents, including information regarding admissions, discharges, transfers to hospital, and readmissions to nursing homes. It also includes information on Medicare coverage for postacute care for each resident. These files were merged with the CMS Impact File, which includes data on hospital characteristics (number of beds, average daily census, resident-to-bed ratio, disproportionate share hospital percentage [DSH], and geographic region).

### Study Population

We identified 4 970 875 community-dwelling individuals hospitalized for sepsis between January 1, 2016, and December 31, 2021 (eFigure 1 in Supplement 1). Patients were defined as community-dwelling prior to the index sepsis hospital admission if they resided at least 30 continuous days in the community prior to admission. Cases of sepsis were identified following the methodology defined by Rudd and colleagues^16^ in the Global Burden of Disease Study using *ICD-10* codes referencing sepsis explicitly (eg, R65.2, severe sepsis). The Research Triangle Institute codes were used to categorize race and ethnicity.^17^ We excluded patients younger than 65 years of age (970 734), discharged after June 1, 2021 (203 870 [these patients were excluded to allow for sufficient follow-up]), race unknown or other (63 891), in-hospital death or discharge to hospice (590 951), or hospitalizations in hospitals not included in the CMS Impact File (11 376). The final analytic dataset consisted of 2 964 517 observations in 3209 hospitals.

### Statistical Analysis

The outcome of interest was the long-term NH stay or death. Patients discharged alive experienced long-term NH stay or death if they resided in a nursing home 101 days after hospital discharge and had no period at home greater than 30 days, or died 30 or more days following hospital discharge. We chose 101 days as the start of a long-term nursing home care stay because Medicare only pays for short-term stays that are 100 days or less.

We used interrupted time-series analysis to examine whether the pandemic was associated with changes in long-term NH stay or death in patients discharged alive following a sepsis hospitalization, and whether these changes varied by race and ethnicity. We estimated the multivariable logistic regression model shown here:

*f*[*E*(*Y_iht_*)] = β_0_* *+* *β_1_*_k_RaceEthnic_k_ *+* *β_2_*Time *+* *β_3_*Post *+* *β_4_*TimeCOVID *+* *β_5_*_k_Post × RaceEthnic_k_ *+* *β_6_*_k_TimeCOVID × RaceEthnic_k_ *+* *β_7_*_k_X_k_ *+* *β_8_*_k_Z_k_ *+* *β_9_*_k_Month_k_*

where *f* is the logit function, *Y_ikt_* is long-term NH stay or death for patient *i* in hospital *k* at time *t*; *RaceEthnic_k_* is a categorical variable for race and ethnicity (American Indian or Alaska Native, Asian or Pacific Islander, Black, Hispanic, and White). *Time* is the underlying quarterly (3-month) time trend; *Post* is an indicator variable for the pandemic and is 0 before April 1, 2020, and 1 after; *TimeCOVID* is the superimposed quarterly time trend starting in April 2020; and *Month_k_* is a vector of monthly indicators to identify seasonal variation. The interaction terms between (1) *Post* and *RaceEthnic* and (2) *TimeCOVID* and *RaceEthnic* quantify the additional effect of the pandemic on minoritized individuals compared with White individuals. *X_k_* is a vector of patient-level characteristics (demographics [age, sex], frailty [specified separately as malnutrition, senility, cognitive dysfunction, urinary incontinence, fecal incontinence, gait disturbance, dependent on caregiver], social determinants of health [housing instability, social environment], prior myocardial infarction [MI: prior ST-segment elevation MI, non-ST segment elevation MI, other MI], pulmonary disease [acute respiratory distress syndrome, pulmonary edema, pulmonary interstitial edema, acute respirtory failure, acute on chronic respiratory failure, chronic respiratory failure, respiratory failure unspecified], COVID-19, individual Elixhauser comorbidities,^18^ prior procedures [percutaneous coronary intervention, coronary artery bypass graft surgery, heart valve surgery, left ventricular assist device, kidney transplant], a categorial variable for use of intensive care [none, step-down unit, intensive care unit], and in-hospital complications [myocardial infarction, congestive heart failure, stroke, acute renal failure, respiratory failure]) (eTable 1 in Supplement 1). We used the present-on-admission indicator to differentiate between complications and preexisting conditions. *Z_k_* is a vector of hospital characteristics (number of beds, disproportionate share hospital [DSH] patient percentage [a higher DSH patient percentage indicates a greater proportion of low-income patients], resident-to-bed ratio, average daily census, geographic region, and the hospital proportion of Black patients with sepsis).

We estimated a series of sequential models to better understand the potential association of structural racism and hospital factors with long-term NH stay or death. First, we estimated a baseline model that only adjusted for patient demographics because minoritized individuals may present with greater multimorbidity than White individuals because of the effect of social determinants of health and structural racism. We then estimated a series of models in which we added (1) comorbidities, (2) in-hospital complications, and (3) hospital characteristics because these factors may decrease the size of the association between the pandemic and changes in long-term NH stay or death for minoritized individuals. Finally, we performed 2 post hoc analyses that were suggested by peer reviewers. In the first, we changed the definition of long-term NH stay or death to exclude patients who died 100 days or fewer following hospital discharge to examine long-term NH stay or death only attributable to long-term care. In the second, we excluded patients with COVID-19.

All statistical analyses were performed using STATA/MP version 18.0 (StataCorp) from May to November 2024. We used a cluster robust variance estimator to account for the clustering of observations within hospitals. We estimated adjusted rates, adjusted rate differences, and average marginal effects. The threshold for statistical significance was 2-sided *P* < .05.

## Results

### Patient Population

Among the 2 964 517 hospitalizations for sepsis of community-dwelling patients discharged alive, 1 468 754 (49.5%) were female; 19 549 (0.7%) American Indian or Alaska Native, 95 308 (3.2%) Asian or Pacific Islander, 282 646 (9.5%) Black, 279 011 (9.4%) Hispanic, and 2 288 003 (71.2%) White individuals; the mean (SD) age was 76 (8.3) years (eTable 2 in Supplement 1). Black (15 384 [5.4%]) and Hispanic (16 218 [5.8%]) individuals were more likely to have COVID-19 compared with White individuals (51 900 [2.3%]). Asian or Pacific Islander (10 448 [11.0%]) and Black (39 605 [14.0%]) individuals were more likely to have a history of severe kidney failure than White (132 579 [5.8%]) individuals. Asian or Pacific Islander (33 678 [35.3%]), Black (81 164 [29.4%]), Hispanic (117 561 [42.1%]), and American Indian or Alaska Native (6570 [33.6%]) individuals were more likely to be hospitalized in hospitals with disproportionate share percentage 50% or greater compared with White (506 054 [22.1%]) individuals. Hospital characteristics are shown in eTable 3 in Supplement 1.

### Association of Race and Ethnicity With Long-Term NH Stay or Death

**Figure 1.**
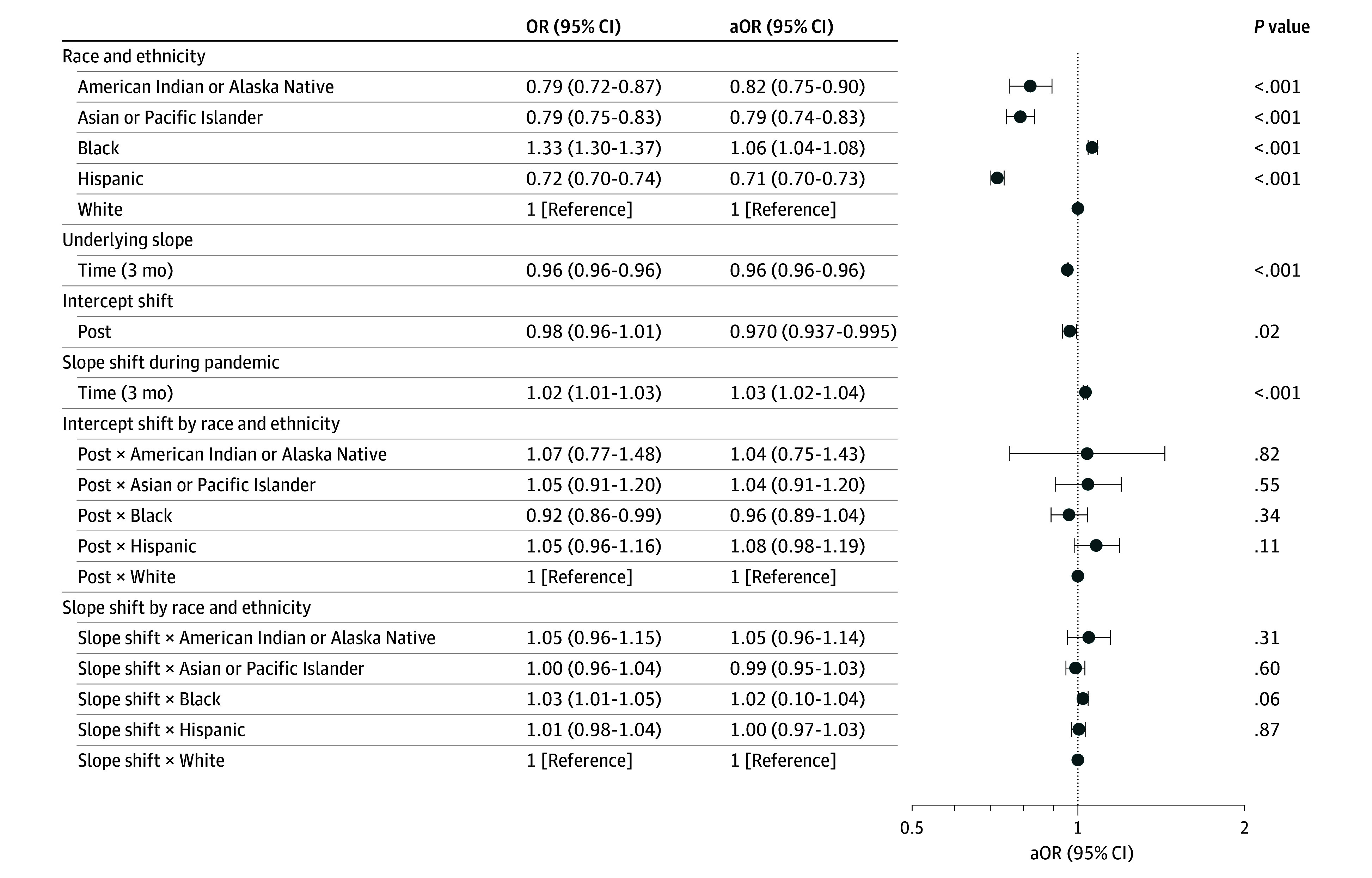
Association Between Long-Term Nursing Home Stay or Death and COVID-19 Pandemic These findings are based on an interrupted time-series model. The unadjusted model is adjusted for age, sex, and race and ethnicity. The adjusted model is adjusted for age, sex, race, ethnicity, comorbidities, in-hospital complications, and hospital characteristics (see Methods). aOR indicates adjusted odds ratio; OR, odds ratio.

During the prepandemic period, after controlling only for age and sex, Black individuals were more likely to experience long-term NH stay or death (adjusted odds ratio [aOR], 1.33; 95% CI, 1.30 to 1.37; *P* < .001) (adjusted risk difference, 2.65%; 95% CI, 2.43% to 2.87%; *P* < .001) compared with White individuals, whereas American Indian or Alaska Native (aOR, 0.79; 95% CI, 0.72 to 0.87; *P* < .001) (adjusted risk difference, −1.50%; 95% CI, −2.11% to −0.89%; *P* < .001), Asian or Pacific Islander (aOR, 0.79; 95% CI, 0.75 to 0.83; *P* < .001) (adjusted risk difference, −1.75%; 95% CI, −2.07% to −1.42%; *P* < .001), and Hispanic (aOR, 0.72; 95% CI, 0.70 to 0.74; *P* < .001) (adjusted risk difference, −2.28%; 95% CI, −2.48% to −2.07%; *P* < .001) individuals were less likely to experience long-term NH stay or death compared with White individuals (adjusted prediction, 9.13%; 95% CI, 9.02% to 9.24%) (Figure 1; eTable 4 in Supplement 1). After adjusting for comorbidities, in-hospital complications, and hospital characteristics, these findings were essentially unchanged, with the exception that the effect size for Black individuals was smaller (aOR, 1.06; 95% CI, 1.04-1.08; *P* < .001) (Figure 1; eTable 4 in Supplement 1).

**Figure 2.**
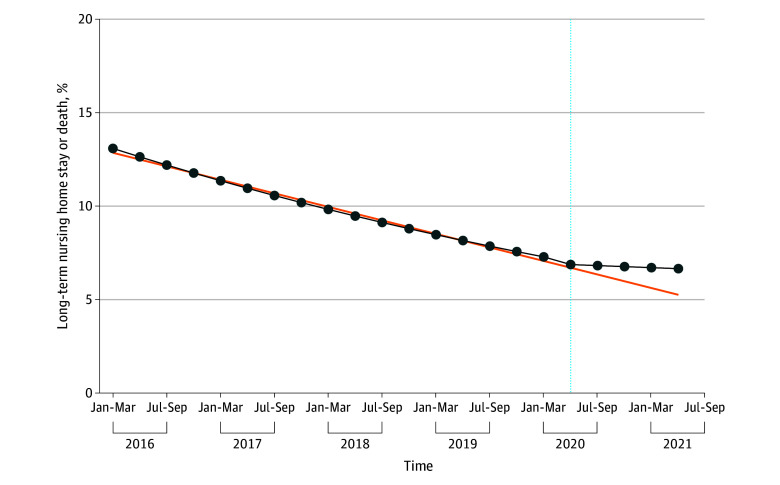
Estimated Long-Term Nursing Home Stay or Death These findings are based on the interrupted series analysis after adjusting for race and ethnicity, patient demographics, frailty, social determinants of health, comorbidities, COVID-19, prior procedures, in-hospital complications, and hospital characteristics. The counterfactual represents the estimated underlying time trend in the absence of the pandemic.

**Figure 3.**
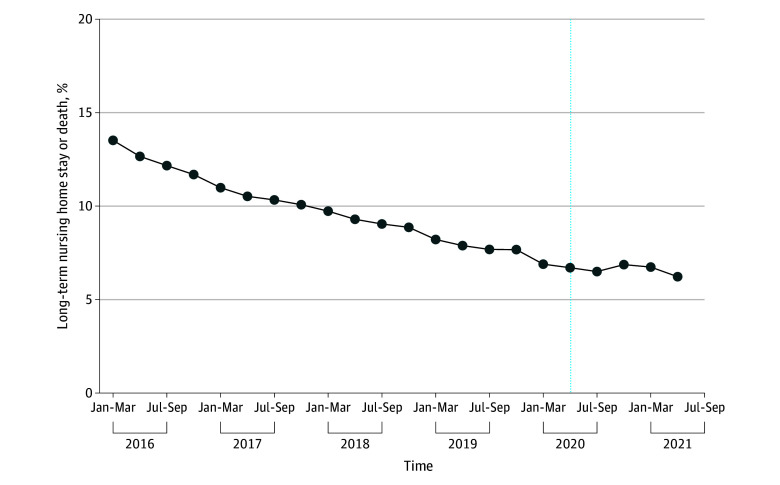
Observed Long-Term Nursing Home Stay or Death

**Figure 4.**
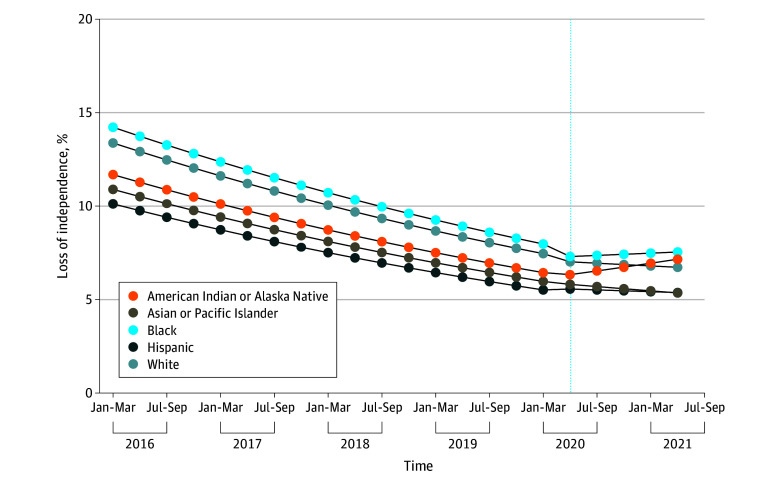
Estimated Long-Term Nursing Home Stay or Death, Stratified by Race and Ethnicity These findings are based on the interrupted series analysis after adjusting for patient demographics, frailty, social determinants of health, comorbidities, COVID-19, prior procedures, in-hospital complications, and hospital characteristics.

Long-term NH stay or death declined from 13.5% in the first quarter of 2016 to 6.9% in the first quarter of 2020. After adjusting for comorbidities, in-hospital complications, and hospital characteristics, the odds of long-term NH stay or death decreased by 4.2% each quarter before the pandemic (aOR, 0.958; 95% CI, 0.957 to 0.959; *P* < .001) (average marginal effect [AME], −0.33%; 95% CI, −0.34% to −0.33%; *P* < .001) (Figures 1 and 2; eTable 4 in Supplement 1). For White individuals, the pandemic was associated with an intercept shift (aOR, 0.97; 95% CI, 0.94 to 0.995; *P* = .02) (AME, −0.24%; 95% CI, −0.46% to −0.04%) and an increase in the risk of long-term NH stay or death over time (aOR, 1.03; 95% CI, 1.02 to 1.04 [each quarter]) (AME, 0.26%; 95% CI, 0.20% to 0.32%) (Figures 1 and 2; eTable 4 in Supplement 1). Accounting for both the prepandemic trend and the change in slope during the pandemic, the postpandemic trend was characterized with a flattening in the risk of long-term NH stay or death (aOR, 0.99; 95% CI, 0.98 to 0.997; *P* = .02). These findings are qualitatively similar with the observed trend in the long-term NH stay or death over time (Figure 3). The pandemic, however, was not associated with significant changes in long-term NH stay or death for minoritized individuals compared with White individuals (Figures 1 and 4; eTable 4 in Supplement 1).

Frailty, social determinants of health, comorbidities, and in-hospital complications were associated with long-term NH stay or death (eTable 4 in Supplement 1). Patients with malnutrition (aOR, 1.47; 95% CI, 1.42-1.52; *P* < .001), fecal incontinence (aOR, 1.51; 95% CI, 1.43-1.61; *P* < .001), and housing instability (aOR, 2.97; 95% CI, 2.74-3.21; *P* < .001) were more likely to experience long-term NH stay or death. Patients with neurologic disorders (aOR, 1.58; 95% CI, 1.56-1.59; *P* < .001), psychoses (aOR, 1.54; 95% CI, 1.50-1.59; *P* < .001), and severe kidney failure (aOR, 1.32; 95% CI, 1.30-1.34; *P* < .001) were also more likely to experience long-term NH stay or death. Finally, patients with in-hospital complications such as congestive heart failure (aOR, 1.40; 95% CI, 1.36-1.43; *P* < .001), stroke (aOR, 2.00; 95% CI, 1.90-2.10; *P* < .001), and acute respiratory failure (aOR, 1.35; 95% CI, 1.33-1.38; *P* <.001) were also more likely to experience long-term NH stay or death. The findings of our post hoc analyses, in which (1) we excluded patients who died within 100 days of hospital discharge (eFigure 2 in Supplement 1) and (2) excluded patients with COVID-19 (eFigure 3 in Supplement 1) were similar to our main findings (Figure 1).

## Discussion

Using national data on 2 964 517 community-dwelling Medicare beneficiaries hospitalized with sepsis between 2016 and 2021, we found that Black individuals had a 33% higher risk of long-term NH stay or death following hospital discharge while Asian or Pacific Islander, Hispanic, and American Indian or Alaska Native individuals had between 21 to 28% lower risk of long-term NH stay or death compared with White individuals. Among all individuals, there was a striking reduction in long-term NH stay or death over time before the pandemic. Long-term NH stay or death declined from 13.5% in the first quarter of 2016 to 6.9% in the first quarter of 2020, but progress slowed markedly during the pandemic. During the pandemic, all individuals experienced similar increases in the rate of long-term NH stay or death compared with the prepandemic trend, regardless of race and ethnicity. These findings remained unchanged following comprehensive risk adjustment.

Our first major finding was the significant racial differences in long-term NH stay or death across the study period, with Black individuals, in particular, much more likely to experience long-term nursing home utilization or death following discharge from a sepsis hospitalization. Prior studies in this space have been mixed. Some have found that White individuals, in contrast with people from other racial or ethnic groups, were more likely to utilize long-term care^19^ and have a lower burden of family caregiving,^20^ perhaps due to cultural differences or differences in access to long-term care insurance or other resources. Research has also demonstrated a relationship between higher skilled nursing home care quality and lower rates of institutionalization,^21^ and people from racial and ethnic minoritized groups received care from lower-quality nursing facilities,^22^ which may also drive these outcomes.

More broadly, our findings also found an approximately 50% reduction in long-term NH stay or death over a 5-year period before the pandemic. This decrease, before the pandemic, in the number of older adults likely to spend the remainder of their lives in a nursing home may be due to several factors, and likely reflects both positive and negative changes in this clinical setting.^23^ On the positive side, this trend may be driven by the growth in availability and sophistication of home and community-based services, increased use of assisted living facilities, and shifts in the preferences of aging individuals; changes that collectively lead to reductions in long-term NH stay or death after a critical illness.^24,25,26,27,28^ In particular, state initiatives increase support for the more cost-effective HCBS, and spending on HCBS has increased from 18% to 57% of spending on long-term care.^24,25^ Furthermore, changes in payment system for Medicare home health services, which were adopted in 2020, may have also promoted the use of HCBS as an alternative to long-term institutionalization by improving the alignment of reimbursements with patient needs.^29^ On the other hand, some of the decrease in long-term care use may reflect unmet needs driven by cost and quality concerns in this industry; there is certainly concern for systemic failures of nursing homes to provide adequate support for those patients whose physical and cognitive disabilities make them suitable for this level of care.^30^ For example, for approximately 90% of elderly individuals, the annual cost of private nursing home care, which can provide individuals with the ability to access higher-quality long-term care, is more than their yearly income.^26^ Quality in long-term care is highly uneven; nearly two-thirds of staff working in institutional settings have admitted to elder abuse.^28^ Not all individuals needing long-term care will have the family or economic resources to age in-home, and some will require institutional long-term care, especially when around-the-clock care is necessary. As such, nursing homes have been described as a “critical safety net for frail older adults,” and ensuring their availability when needed is crucially important.^31^

Unfortunately, we also found that the decreasing rate of long-term NH stay or death plateaued during the COVID-19 pandemic. Given that shifts in preferences away from institutional elder care were likely reinforced by the devastating impact of the pandemic on individuals living in nursing homes,^3,27^ and ongoing supply constraints in nursing facilities as the industry struggles to return to prepandemic staffing levels, the plateauing is even more striking. One possibility for this is the effect of COVID-19 itself; COVID-19 had profound effects on both morbidity and mortality among older adults, and it could have worsened health enough on a broad scale to preclude a significant proportion of people from living safely at home.^32^ Another possibility is a reduction in the availability of alternatives to long-term nursing care, including home-based and community services.^33^ Much like the nursing home industry, this area of care delivery has had difficulty rebounding from COVID-19 in terms of workforce, and many small agencies closed during COVID-19 due to financial strain. It will be crucial to continue to watch trends in this space closely, to determine where capacity must be most urgently addressed to ensure adequate care delivery to at-risk seniors.

Previous studies have documented the pandemic’s disproportionate impact on mortality among minoritized individuals. During the initial wave of the pandemic, Non-Hispanic Black and Hispanic individuals experienced 3.4-fold and 2.2-fold higher mortality rates than Non-Hispanic White individuals, respectively.^34^ In the present study, we found that while all individuals experienced slight increases in long-term NH stay or death during the pandemic compared with before the pandemic, minoritized racial and ethnic groups did not experience greater increases in long-term NH stay or death than White individuals. This is consistent with other studies that did not demonstrate increases in disparities during the pandemic once patients encountered the health care system, such as those undergoing surgery or admitted for acute myocardial infarction, strokes, or COVID-19.^34,35,36,37,38,39^

The devastating impact of the pandemic on nursing homes, which have been described as “ground zero” for the pandemic, has prompted renewed efforts to address quality issues in nursing homes.^30,31^ According to a recent report from the National Academy of Sciences Committee on the Quality of Care in Nursing Homes, the response to the pandemic’s impact on older adults living in nursing homes reflects “pervasive ageism” and the “undervaluing of the lives of older adults.”^30^ Through fundamental change envisioned by the Committee, future decreases in long-term NH stay or death may be more likely to represent a positive outcome as opposed to a possible failure of the safety net for older US residents.

### Limitations

This study has limitations. First, we restricted this study to older community-dwelling individuals hospitalized due to sepsis. Our findings may not be generalizable to individuals younger than 65 years, those hospitalized with other serious medical conditions, or individuals who lose independence without a prior hospitalization. Nonetheless, our population-based study includes a high-risk cohort of patients hospitalized with severe illness requiring resource-intensive care. Second, our study may have been underpowered to examine changes in the long-term NH stay or death for American Indian or Alaska Native and Asian or Pacific Islander individuals. We included Americans Indian or Alaska Native individuals because they represent an at-risk and relatively understudied population who have been disproportionately affected by the pandemic. Third, our definition of long-term NH stay or death is based on the maximum length of Medicare coverage for postacute care in skilled nursing facilities. Although this numeric cutoff is arbitrary, we assumed that patients who did not require long-term nursing care would be financially incentivized to return to the community by this Medicare payment policy. Fourth, the accuracy of race and ethnicity data in Medicare is high for Black and White individuals, but is much lower for other racial and ethnic categories.^40^ However, we instead used the Research Triangle Institute (RTI) race and ethnicity code in the Medicare data, which is based on an imputation algorithm that markedly improves the sensitivity for coding of Hispanic and Asian and Pacific Islander individuals.^15^ Additionally, although we adjusted for a comprehensive list of patient risk factors, this study is based on Medicare claims data instead of clinical data, which could lead to unmeasured confounding. However, in order for unmeasured confounding to have caused the impact of the pandemic on minoritized individuals to be downwardly biased, it woud have been necessary for minoritized individuals to be more likely to have unmeasured risk factors that decrease the risk of long-term NH stay or death compared with White individuals; this is unlikely because many social determinants of health such as social support, which are not well captured in claims data, are associated with increases and not decreases in the need for long-term nursing home care.

## Conclusions

In this cross-sectional study, we found that over the 5-year period before the pandemic, community-dwelling older adults hospitalized with sepsis experienced an approximately 50% reduction in long-term NH stay or death. Black individuals experienced a greater long-term NH stay or death while Hispanic and other minoritized individuals experienced less long-term NH stay or death compared with White individuals prior to the pandemic. Among all individuals, regardless of race and ethnicity, decreases in long-term NH stay or death slowed during the pandemic. The renewed focus on nursing homes during the pandemic presents an opportunity to strengthen nursing homes, which serve as the safety net for the most at-risk older individuals.

## Notes

Supplement 1. **eFigure 1.** Study Flow Diagram for Analytic Cohort **eFigure 2.** Association Between Long-Term Nursing Home Stay or Death and COVID-19 Pandemic Excluding Patients Who Died Within 100 Days of Hospital Discharge **eFigure 3.** Association Between Long-Term Nursing Home Stay or Death and COVID-19 Pandemic excluding patients with COVID-19 **eTable 1.** ICD-10-CM Diagnostic Codes **eTable 2.** Patient Characteristics **eTable 3.** Hospital Characteristics **eTable 4.** Regression Models

Supplement 2.
Data Sharing Statement

## References

[1] 2023 National population projections tables: main series (United States Census Bureau). Published 2024. Accessed April 24, 2024. https://www.census.gov/data/tables/2023/demo/popproj/2023-summary-tables.html

[2] Butler SM. The challenging future of long-term care for older adults. JAMA Health Forum. 2022;3(5):e222133. doi:10.1001/jamahealthforum.2022.2133 36219037

[3] McGarry BE, Grabowski DC. Nursing homes and COVID-19: a crisis on top of a crisis. Ann Am Acad Polit Ss. 2021;698(1):137-162. doi:10.1177/00027162211061509

[4] Sloane PD, Yearby R, Konetzka RT, Li Y, Espinoza R, Zimmerman S. Addressing systemic racism in nursing homes: a time for action. J Am Med Dir Assoc. 2021;22(4):886-892. doi:10.1016/j.jamda.2021.02.023 33775548

[5] CMS. The impact of COVID-19 on Medicare on Medicare beneficiaries in nursing homes. Accessed April 24, 2024. https://www.google.com/url?sa=t&source=web&rct=j&opi=89978449&url=https://www.cms.gov/files/document/medicare-covid-19-nursing-home-analysis.pdf&ved=2ahUKEwjy6aa31tuFAxXCElkFHe5iCs8QFnoECBgQAQ&usg=AOvVaw3laqJN6jUrRZEocvsYkLyW

[6] Gorges RJ, Konetzka RT. Factors associated with racial differences in deaths among nursing home residents with COVID-19 infection in the US. JAMA Netw Open. 2021;4(2):e2037431. doi:10.1001/jamanetworkopen.2020.37431 33566110 PMC7876590

[7] Berian JR, Mohanty S, Ko CY, Rosenthal RA, Robinson TN. Association of loss of independence with readmission and death after discharge in older patients after surgical procedures. JAMA Surg. 2016;151(9):e161689. doi:10.1001/jamasurg.2016.1689 27409710

[8] Blank M, Robitaille MJ, Wachtendorf LJ, . Loss of independent living in patients undergoing transcatheter or surgical aortic valve replacement: a retrospective cohort study. Anesth Analg. 2023;137(3):618-628. doi:10.1213/ANE.0000000000006377 36719955

[9] Werner RM, Bressman E. Trends in post-acute care utilization during the COVID-19 pandemic. J Am Med Dir Assoc. 2021;22(12):2496-2499. doi:10.1016/j.jamda.2021.09.001 34555340 PMC8421095

[10] Ulyte A, Waken RJ, Epstein AM, . Medicare skilled nursing facility use and spending before and after introduction of the public health emergency waiver during the COVID-19 pandemic. JAMA Intern Med. 2023;183(7):637-645. doi:10.1001/jamainternmed.2023.0770 37093607 PMC10126940

[11] Prest J, Sathananthan M, Jeganathan N. Current trends in sepsis-related mortality in the United States. Crit Care Med. 2021;49(8):1276-1284. doi:10.1097/CCM.0000000000005017 34261926

[12] Centers for Disease Control and Prevention. What is sepsis? Published 2024. Accessed February 26, 2024. https://www.cdc.gov/sepsis/about/index.html

[13] Wang HE, Kabeto MM, Gray M, . Trajectory of cognitive decline after sepsis. Crit Care Med. 2021;49(7):1083-1094. doi:10.1097/CCM.0000000000004897 33666392 PMC8217073

[14] von Elm E, Altman DG, Egger M, Pocock SJ, Gøtzsche PC, Vandenbroucke JP; STROBE Initiative. The Strengthening the Reporting of Observational Studies in Epidemiology (STROBE) statement: guidelines for reporting observational studies. Lancet. 2007;370(9596):1453-1457. doi:10.1016/S0140-6736(07)61602-X 18064739

[15] Eicheldinger C, Bonito A. More accurate racial and ethnic codes for Medicare administrative data. Health Care Financ Rev. 2008;29(3):27-42. 18567241 PMC4195038

[16] Rudd KE, Johnson SC, Agesa KM, . Global, regional, and national sepsis incidence and mortality, 1990-2017: analysis for the Global Burden of Disease Study. Lancet. 2020;395(10219):200-211. doi:10.1016/S0140-6736(19)32989-7 31954465 PMC6970225

[17] Research Triangle Institute (RTI) Race Code. Accessed October 2022. https://resdac.org/cms-data/variables/research-triangle-institute-rti-race-code

[18] AHRQ. Elixhauser comorbidity software refined for ICD-10-CM. Accessed April 13, 2023. https://hcup-us.ahrq.gov/toolssoftware/comorbidityicd10/comorbidity_icd10.jsp#:~:text=The%20Elixhauser%20Comorbidity%20Indices%20Refined%20for%20ICD%2D10%2DCM%20is,%2Dday%2C%20all%2Dcause%20readmission

[19] Middleton A, Li S, Kuo YF, Ottenbacher KJ, Goodwin JS. New institutionalization in long-term care after hospital discharge to skilled nursing facility. J Am Geriatr Soc. 2018;66(1):56-63. doi:10.1111/jgs.15131 29112226 PMC5777887

[20] Cantu P, Cho TC, Wyman M, Helppie-McFall B, Ajrouch KJ. Racial and ethnic disparities in the monetary value of informal caregiving for non-institutionalized people living with dementia. J Aging Health. 2024;36(9):570-582. doi:10.1177/08982643241262917 38887015 PMC11363470

[21] Goodwin JS, Li S, Middleton A, Ottenbacher K, Kuo YF. Differences between skilled nursing facilities in risk of subsequent long-term care placement. J Am Geriatr Soc. 2018;66(10):1880-1886. doi:10.1111/jgs.15377 29656399 PMC6181774

[22] Zuckerman RB, Wu S, Chen LM, Joynt Maddox KE, Sheingold SH, Epstein AM. The five-star skilled nursing facility rating system and care of disadvantaged populations. J Am Geriatr Soc. 2019;67(1):108-114. doi:10.1111/jgs.15629 30339726

[23] Werner RM, Hoffman AK, Coe NB. Long-term care policy after Covid-19 - solving the nursing home crisis. N Engl J Med. 2020;383(10):903-905. doi:10.1056/NEJMp2014811 32459918

[24] CMS. Medicaid Long Term Services and Supports Annual Expenditures Report. Accessed June 24, 2024. https://www.mathematica.org/publications/medicaid-long-term-services-and-supports-annual-expenditures-report-federal-fiscal-year-2019

[25] Grabowski DC. The future of long-term care requires investment in both facility- and home-based services. Nat Aging. 2021;1(1):10-11. doi:10.1038/s43587-020-00018-y 37117999

[26] McGarry JGKM. Long-term care in the United States. Accessed June 24, 2024. https://www.nber.org/papers/w31881

[27] Achou B, De Donder P, Glenzer F, Lee M, Leroux ML. Nursing home aversion post-pandemic: Implications for savings and long-term care policy. J Econ Behav Organ. 2022;201:1-21. doi:10.1016/j.jebo.2022.06.034 35891625 PMC9303513

[28] Yon Y, Ramiro-Gonzalez M, Mikton CR, Huber M, Sethi D. The prevalence of elder abuse in institutional settings: a systematic review and meta-analysis. Eur J Public Health. 2019;29(1):58-67. doi:10.1093/eurpub/cky093 29878101 PMC6359898

[29] Riekert S. Patient-Driven Groupings Model (PDGM). Home Healthc Now. 2019;37(5):300. doi:10.1097/NHH.0000000000000808 31483367

[30] The National Imperative to Improve Nursing Home Quality. Honoring Our Commitment to Residents, Families, and Staff. In: National Academies of Sciences, Engineering, and Medicine, ed. Washington, DC: The National Academies Press; 2022. doi:10.17226/26526 36198022

[31] Barnett ML, Grabowski DC. Nursing homes are ground zero for COVID-19 pandemic. JAMA Health Forum. 2020;1(3):e200369. doi:10.1001/jamahealthforum.2020.0369 36218601

[32] Koff WC, Williams MA. Covid-19 and immunity in aging populations - a new research agenda. N Engl J Med. 2020;383(9):804-805. doi:10.1056/NEJMp2006761 32302079

[33] KFF. Pandemic-era changes to Medicaid home- and community-based services (HCBS). a closer look at family caregiver policies. Accessed July 2, 2024. https://www.kff.org/medicaid/issue-brief/pandemic-era-changes-to-medicaid-home-and-community-based-services-hcbs-a-closer-look-at-family-caregiver-policies/

[34] Lundberg DJ, Wrigley-Field E, Cho A, . COVID-19 mortality by race and ethnicity in US metropolitan and nonmetropolitan areas, March 2020 to February 2022. JAMA Netw Open. 2023;6(5):e2311098. doi:10.1001/jamanetworkopen.2023.11098 37129894 PMC10155069

[35] Glance LG, Dick AW, Shippey E, . Association between the COVID-19 pandemic and insurance-based disparities in mortality after major surgery among US adults. JAMA Netw Open. 2022;5(7):e2222360. doi:10.1001/jamanetworkopen.2022.22360 35849395 PMC9294995

[36] Glance LG, Joynt Maddox KE, Shang J, . The COVID-19 pandemic and associated inequities in acute myocardial infarction treatment and outcomes. JAMA Netw Open. 2023;6(8):e2330327. doi:10.1001/jamanetworkopen.2023.30327 37624599 PMC10457721

[37] Glance LG, Benesch CG, Joynt Maddox KE, . Was COVID-19 associated with worsening inequities in stroke treatment and outcomes? J Am Heart Assoc. 2023;12(19):e031221. doi:10.1161/JAHA.123.031221 37750574 PMC10727248

[38] Glance LG, Maddox KEJ, Mazzeffi M, . Insurance-based disparities in outcomes and extracorporeal membrane oxygenation utilization for hospitalized COVID-19 patients. Anesthesiology. 2024;141(1):116-130. doi:10.1097/ALN.0000000000004985 PMC11318453 38526387

[39] Price-Haywood EG, Burton J, Fort D, Seoane L. Hospitalization and mortality among Black patients and White patients with Covid-19. N Engl J Med. 2020;382(26):2534-2543. doi:10.1056/NEJMsa2011686 32459916 PMC7269015

[40] Filice CE, Joynt KE. Examining race and ethnicity information in Medicare administrative data. Med Care. 2017;55(12):e170-e176. doi:10.1097/MLR.0000000000000608 29135782

