# Supplementary material for: COVID-19 Pandemic and Racial and Ethnic Disparities in Long-Term Nursing Home Stay or Death Following Hospital Discharge
Source: JAMA Netw Open. 2025 Jan 24;8(1):e2456816. doi: 10.1001/jamanetworkopen.2024.56816 (PMC11762228; doi:10.1001/jamanetworkopen.2024.56816)
Supplement: Supplement 1. — eFigure 1. Study Flow Diagram for Analytic Cohort eFigure 2. Association Between Long-Term Nursing Home Stay or Death and COVID-19 Pandemic Excluding Patients Who Died Within 100 Days of Hospital Discharge eFigure 3. Association Between Long-Term Nursing Home Stay or Death and COVID-19 Pandemic excluding patients with COVID-19 eTable 1. ICD-10-CM Diagnostic Codes eTable 2. Patient Characteristics eTable 3. Hospital Characteristics eTable 4. Regression Models [file jamanetwopen-e2456816-s001.pdf]

Supplemental Online Content

Glance LG, Joynt Maddox KE, Stone PW, et al. COVID-19 pandemic and racial and ethnic disparities in long-term nursing home stay or death following hospital discharge. *JAMA Netw Open.* 2025;8(1):e2456816. doi:10.1001/jamanetworkopen.2024.56816

eFigure 1. Study Flow Diagram for Analytic Cohort

eFigure 2. Association Between Long-Term Nursing Home Stay or Death and COVID-19 Pandemic Excluding Patients Who Died Within 100 Days of Hospital Discharge

eFigure 3. Association Between Long-Term Nursing Home Stay or Death and COVID-19 Pandemic excluding patients with COVID-19

eTable 1. ICD-10-CM Diagnostic Codes

eTable 2. Patient Characteristics

eTable 3. Hospital Characteristics

eTable 4. Regression Models

This supplemental material has been provided by the authors to give readers additional information about their work.

© 2025 Glance LG et al. *JAMA Network Open*.

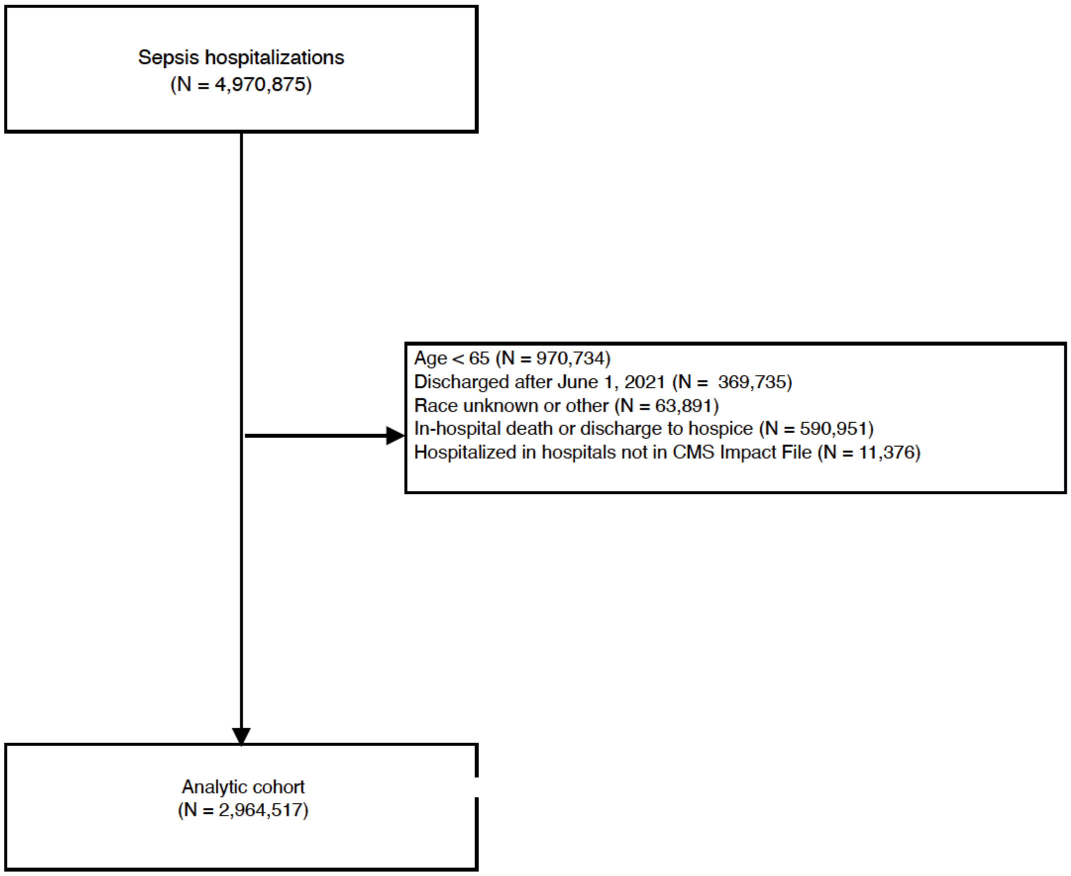

Supplement Figure 1. Study flow diagram for analytic cohort.

© 2025 Glance LG et al. *JAMA Network Open*.

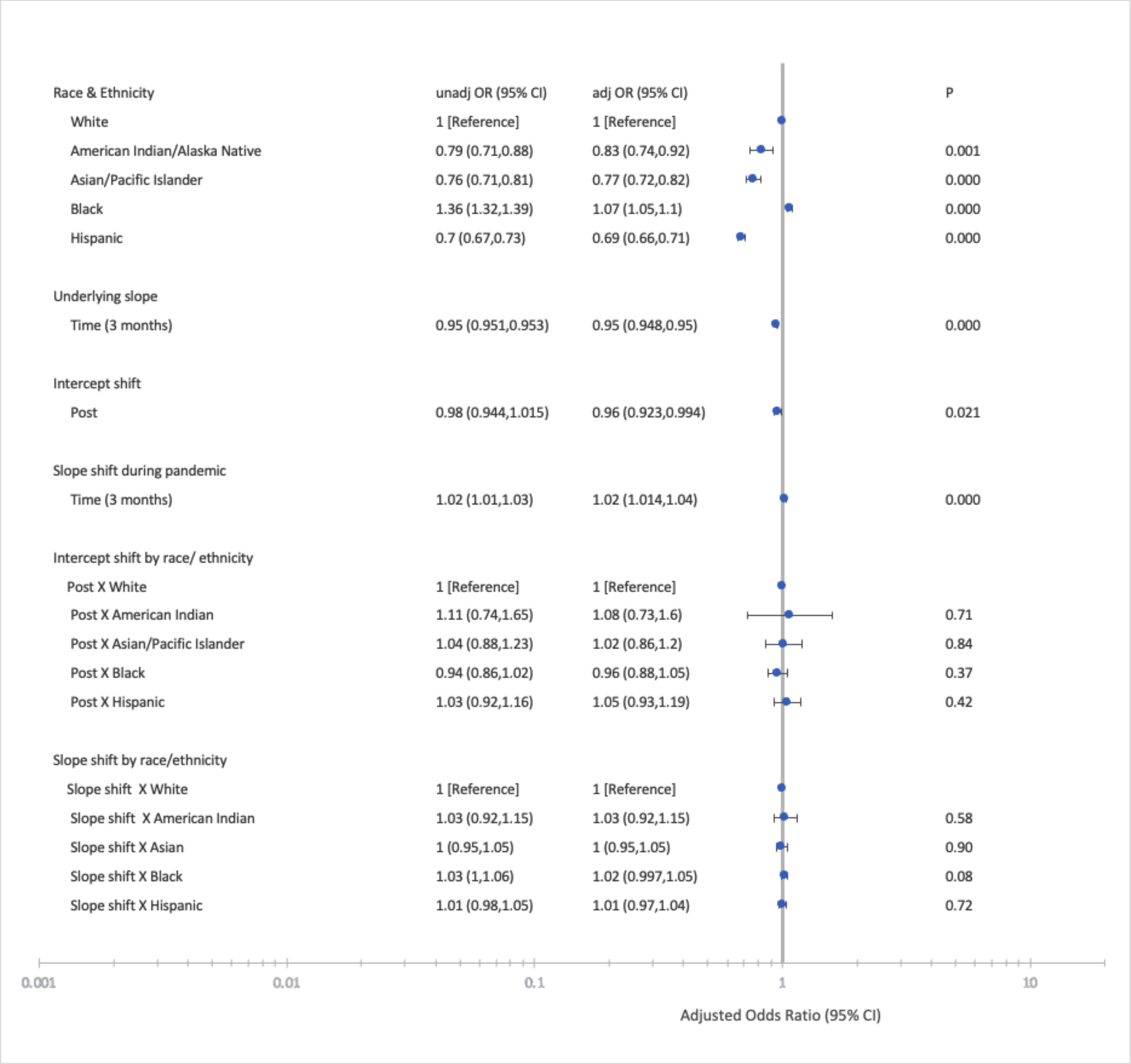

Race & Ethnicity	unadj OR (95% CI)	adj OR (95% CI)	P
White	1 [Reference]	1 [Reference]	
American Indian/Alaska Native	0.79 (0.71,0.88)	0.83 (0.74,0.92)	0.001
Asian/Pacific Islander	0.76 (0.71,0.81)	0.77 (0.72,0.82)	0.000
Black	1.36 (1.32,1.39)	1.07 (1.05,1.1)	0.000
Hispanic	0.7 (0.67,0.73)	0.69 (0.66,0.71)	0.000
Underlying slope			
Time (3 months)	0.95 (0.951,0.953)	0.95 (0.948,0.95)	0.000
Intercept shift			
Post	0.98 (0.944,1.015)	0.96 (0.923,0.994)	0.021
Slope shift during pandemic			
Time (3 months)	1.02 (1.01,1.03)	1.02 (1.014,1.04)	0.000
Intercept shift by race/ ethnicity			
Post X White	1 [Reference]	1 [Reference]	
Post X American Indian	1.11 (0.74,1.65)	1.08 (0.73,1.6)	0.71
Post X Asian/Pacific Islander	1.04 (0.88,1.23)	1.02 (0.86,1.2)	0.84
Post X Black	0.94 (0.86,1.02)	0.96 (0.88,1.05)	0.37
Post X Hispanic	1.03 (0.92,1.16)	1.05 (0.93,1.19)	0.42
Slope shift by race/ethnicity			
Slope shift X White	1 [Reference]	1 [Reference]	
Slope shift X American Indian	1.03 (0.92,1.15)	1.03 (0.92,1.15)	0.58
Slope shift X Asian	1 (0.95,1.05)	1 (0.95,1.05)	0.90
Slope shift X Black	1.03 (1,1.06)	1.02 (0.997,1.05)	0.08
Slope shift X Hispanic	1.01 (0.98,1.05)	1.01 (0.97,1.04)	0.72

Supplement Figure 2. Association Between Long-term Nursing Home Stay or Death and COVID-19 Pandemic excluding patients who died within 100 days of hospital discharge.

These findings are based on an interrupted time-series model. The *unadjusted model* is adjusted for age, sex, and race and ethnicity. The *adjusted model* is adjusted for age, sex, race, ethnicity, comorbidities, in-hospital complications, and hospital characteristics (see Methods).

© 2025 Glance LG et al. *JAMA Network Open*.

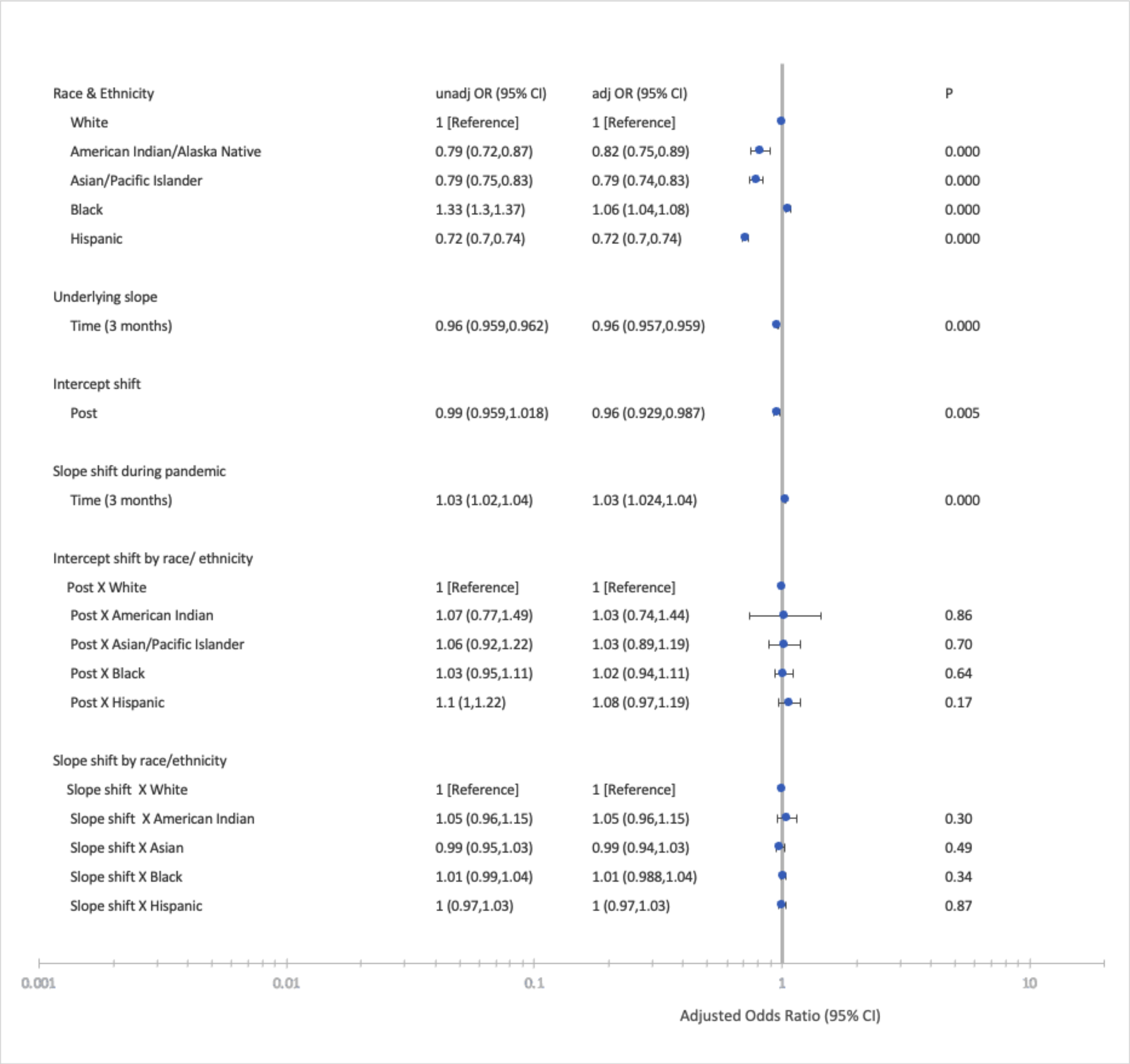

Supplement Figure 3. Association Between Long-term Nursing Home Stay or Death and COVID-19 Pandemic excluding patients with COVID-19.

These findings are based on an interrupted time-series model. The *unadjusted model* is adjusted for age, sex, and race and ethnicity. The *adjusted model* is adjusted for age, sex, race, ethnicity, comorbidities, in-hospital complications, and hospital characteristics (see Methods).

© 2025 Glance LG et al. *JAMA Network Open*.

Supplement Table 1. ICD-10-CM and ICD-10-PCS codes used to identifiy patient characteristics.

Sepsis	
	A02.1; A20.7; A21.7;A22.7; A24.1; A26.7; A28.2; A32.7; A39.4; A40; A41; A42.7; A54.86; B00.7; B37.7; R65,2
**Functional Status/frailty**	
Malnutrition	E43; E44; E45; E46
Senility	R54
Urinary incontinence	R32
Fecal incontinence	R15
Gait disturbane	R262; R268
Cognitive dysfunction	R41
Dependent on provider	Z74
Housing instability	Z59
Social environment	Z60
**Pre-existing Conditions**	
Congestive heart failure	
Systolic CHF	I502
Diastolic CHF	I503
Systolic and diastolic CHF	I504
Unspecified CHF	I501; I509; II5089
Myocardial infarction	
Prior ST-segment elevation MI	I210; I220; I211; I221; I212; I228; I213; I229
Prior Non-ST-segment elevation MI	I214; I222
Prior other MI	I219; I21A
Pulmonary	
Acute respiratory distress syndrome	J80
Pulmonary edema	J81
Pulmonary interstitial disease	J84
Acute respiratory failure	J960
Acute on chronic respiratory failure	J962
Chronic respiratory failure	J961
Respiratory failure, unspecified	J969
COVID-19	U071
**Prior procedures**	
PCI	Z9861; Z955
CABG	Z951
Heart valve surgery	Z952;Z953;Z954
Left ventricular asist device	Z95811
Kidney transplant	Z940
**In-hospital complilcations**	
Acute myocardial infarction	I21; I22
Congestive heart failure	I50
Stroke	I60; I61; I62; I63; I69
Acute renal failure	N17
Acute respiratory failure	J960; J962; J969

© 2025 Glance LG et al. *JAMA Network Open*.

Supplement Table 2. Patient Characteristics.

	Total (N=2,964,517)	White (N=2,288,003)	Black (N=282,646)	Asian (N=95,308)	Hispanic (N=279,011)	Native American/ Alaska Native (N=19,549)
**Characteristics**						
**Age (SD)**	76 (8.3)	76 (8.2)	73 (8.2)	77 (9)	75 (8.4)	74 (7.6)
Sex						
Female	1,468,754 (49.5)	1,121,616 (49)	148,717 (52.6)	46,421 (48.7)	141,020 (50.5)	10,980 (56.2)
**Functional Status/frailty**						
Wheelchair	23,670 (0.8)	17,368 (0.8)	3,038 (1.1)	860 (0.9)	2,222 (0.8)	182 (0.9)
Oxygen supplementation	180,008 (6.1)	153,863 (6.7)	12,056 (4.3)	2,663 (2.8)	10,088 (3.6)	1,338 (6.8)
Malnutrition	339,304 (11.5)	247,580 (10.8)	44,875 (15.9)	13,679 (14.4)	30,910 (11.1)	2,260 (11.6)
Senility	10,130 (0.3)	7,826 (0.3)	1,059 (0.4)	289 (0.3)	875 (0.3)	81 (0.4)
Urinary incontinence	59,773 (2)	47,076 (2.1)	5,887 (2.1)	1,738 (1.8)	4,692 (1.7)	380 (1.9)
Fecal incontinence	9,448 (0.3)	7,399 (0.3)	1,092 (0.4)	258 (0.3)	634 (0.2)	65 (0.3)
Gait disturbane	17,846 (0.6)	14,119 (0.6)	1,736 (0.6)	546 (0.6)	1,347 (0.5)	98 (0.5)
Cognitive dysfunction	59,981 (2)	47,317 (2.1)	5,627 (2)	1,785 (1.9)	4,797 (1.7)	455 (2.3)
Dependent on provider	37,415 (1.3)	20,124 (0.9)	7,497 (2.7)	1,916 (2)	7,720 (2.8)	158 (0.8)
Housing instability	7,330 (0.3)	4,884 (0.2)	1,309 (0.5)	203 (0.2)	786 (0.3)	148 (0.8)
Social environment	9,079 (0.3)	7,409 (0.3)	819 (0.3)	216 (0.2)	574 (0.2)	61 (0.3)
**Pre-existing Conditions**						
Congestive heart failure						
None	2,219,563 (74.9)	1,707,262 (74.6)	205,572 (72.7)	74,763 (78.4)	217,207 (77.9)	14,759 (75.5)
Systolic CHF	183,650 (6.2)	142,442 (6.2)	19,963 (7.1)	4,596 (4.8)	15,455 (5.5)	1,194 (6.1)
Diastolic CHF	332,436 (11.2)	263,267 (11.5)	32,132 (11.4)	9,691 (10.2)	25,412 (9.1)	1,934 (9.9)
Systolic and diastolic CHF	76,759 (2.6)	59,268 (2.6)	8,596 (3)	1,906 (2)	6,389 (2.3)	600 (3.1)
Unspecified CHF	152,109 (5.1)	115,764 (5.1)	16,383 (5.8)	4,352 (4.6)	14,548 (5.2)	1,062 (5.4)
Myocardial infarction						
No prior myocardial infarction	2,793,648 (94.2)	2,156,242 (94.2)	266,669 (94.4)	89,133 (93.5)	263,209 (94.3)	18,395 (94.1)
Prior ST-segment elevation MI	4,019 (0.1)	3,054 (0.1)	387 (0.1)	172 (0.2)	386 (0.1)	20 (0.1)
Prior Non-ST-segment elevation MI	88,108 (3)	67,648 (3)	7,777 (2.8)	3,355 (3.5)	8,792 (3.2)	536 (2.7)
Prior other MI	78,742 (2.7)	61,059 (2.7)	7,813 (2.8)	2,648 (2.8)	6,624 (2.4)	598 (3.1)
Pulmonary						
Acute respiratory distress syndrome	10,401 (0.4)	6,556 (0.3)	1,441 (0.5)	495 (0.5)	1,793 (0.6)	116 (0.6)
Pulmonary edema	29,002 (1)	21,614 (0.9)	2,654 (0.9)	1,271 (1.3)	3,270 (1.2)	193 (1)
Pulmonary interstitial disease	(0)	45,663 (2)	3,802 (1.4)	2,235 (2.4)	6,799 (2.4)	597 (3.1)
Acute respiratory failure	549,256 (18.5)	428,667 (18.7)	49,354 (17.5)	17,956 (18.8)	49,712 (17.8)	3,567 (18.3)
Acute on chronic respiratory failure	202,258 (6.8)	170,633 (7.5)	14,844 (5.3)	3,546 (3.7)	11,760 (4.2)	1,475 (7.6)
Chronic respiratory failure	65,231 (2.2)	55,469 (2.4)	4,790 (1.7)	961 (1)	3,566 (1.3)	445 (2.3)
Respiratory failure, unspecified	21,593 (0.7)	15,880 (0.7)	2,234 (0.8)	890 (0.9)	2,467 (0.9)	122 (0.6)
COVID-19	87,846 (3)	51,900 (2.3)	15,384 (5.4)	3,720 (3.9)	16,218 (5.8)	624 (3.2)

© 2025 Glance LG et al. *JAMA Network Open*.

Supplement Table 2. Patient Characteristics.						
	**Total**	**White**	**Black**	**Asian**	**Hispanic**	**Native American/ Alaska Native**
	(N=2,964,517)	(N=2,288,003)	(N=282,646)	(N=95,308)	(N=279,011)	(N=19,549)
**Characteristics**						
**Elixhauser comorbidities**						
Acquired immune deficiency syndrome	6,836 (0.2)	2,800 (0.1)	2,978 (1.1)	74 (0.1)	960 (0.3)	24 (0.1)
Alcohol abuse	78,317 (2.6)	60,453 (2.6)	9,558 (3.4)	939 (1)	6,418 (2.3)	949 (4.9)
Anemia, nutritional deficiency	780,270 (26.3)	556,326 (24.3)	103,692 (36.7)	30,129 (31.6)	84,393 (30.3)	5,730 (29.3)
Anemia, iron deficiency	21,592 (0.7)	16,016 (0.7)	2,776 (1)	706 (0.7)	1,955 (0.7)	139 (0.7)
Lymphoma	63,409 (2.1)	49,103 (2.2)	6,615 (2.3)	1,885 (2)	5,527 (2)	279 (1.4)
Leukemia	46,561 (1.6)	39,114 (1.7)	3,536 (1.3)	1,072 (1.1)	2,687 (1)	152 (0.8)
Metastatic cancer	122,779 (4.1)	94,377 (4.1)	13,407 (4.7)	4,392 (4.6)	10,027 (3.6)	576 (3)
Solid tumor, in situ	1,117 (0.04)	875 (0.04)	117 (0.04)	38 (0.04)	82 (0.03)	5 (0.03)
Solid tumor, without metstasis	176,842 (6)	139,180 (6.1)	17,084 (6)	5,787 (6.1)	13,916 (5)	875 (4.5)
Cerebrovascular disease	143,431 (4.8)	99,456 (4.4)	22,120 (7.8)	6,284 (6.6)	14,677 (5.3)	894 (4.6)
Coagulopathy	387,021 (13.1)	294,924 (12.9)	35,673 (12.6)	15,199 (16)	38,671 (13.9)	2,554 (13.1)
Dementia	386,598 (13)	281,492 (12.3)	44,875 (15.9)	15,436 (16.2)	42,955 (15.4)	1,840 (9.4)
Depression	355,697 (12)	303,319 (13.3)	18,824 (6.7)	5,171 (5.4)	26,191 (9.4)	2,192 (11.2)
Diabetes with chronic complications	785,466 (26.5)	543,546 (16016)	99,716 (2776)	31,387 (706)	103,539 (1955)	7,278 (139)
Liver disease, mild	141,532 (4.8)	99,504 (4.4)	16,340 (5.8)	6,424 (6.7)	18,012 (6.5)	1,252 (6.4)
Liver disease and failure, moderate to severe	40,371 (1.4)	28,896 (1.3)	2,639 (0.9)	1,398 (1.5)	6,921 (2.5)	517 (2.6)
Neurologic disorder affecting movement	122,164 (4.1)	104,091 (4.6)	5,545 (2)	3,086 (3.2)	8,813 (3.2)	629 (3.2)
Neurologic disorder, other	491,829 (16.6)	375,971 (16.4)	56,785 (20.1)	15,341 (16.1)	40,541 (14.5)	3,191 (16.3)
Seizures and epilepsy	93,358 (3.2)	67,291 (2.9)	14,595 (5.2)	2,162 (2.3)	8,705 (3.1)	605 (3.1)
Obesity	504,814 (17)	396,565 (17.3)	51,392 (18.2)	6,293 (6.6)	47,026 (16.9)	3,538 (18.1)
Paralysis	115,383 (3.9)	74,146 (3.2)	21,516 (7.6)	5,496 (5.8)	13,536 (4.9)	689 (3.5)
Peripheral vascular disease	291,522 (9.8)	224,338 (9.8)	26,709 (9.5)	10,882 (11.4)	27,972 (10)	1,621 (8.3)
Psychoses	76,000 (2.6)	60,359 (2.6)	7,759 (2.8)	1,624 (1.7)	5,825 (2.1)	433 (2.2)
Renal failure, moderate	620,065 (20.9)	480,294 (21)	66,525 (23.5)	19,361 (20.3)	50,453 (18.1)	3,432 (17.6)
Renal failure, severe	215,796 (7.3)	132,579 (5.8)	39,605 (14)	10,448 (11)	30,991 (11.1)	2,173 (11.1)
Peptic ulcer disease with bleeding	45,990 (1.6)	34,707 (1.5)	4,561 (1.6)	2,156 (2.3)	4,283 (1.5)	283 (1.5)
Valvular disease	296,439 (10)	245,638 (10.7)	20,094 (7.1)	8,285 (8.7)	21,032 (7.5)	1,390 (7.1)
Weight loss	354,406 (12)	257,757 (11.3)	47,232 (16.7)	14,594 (15.3)	32,534 (11.7)	2,289 (11.7)
**Prior procedures**						
PCI	214,172 (7.2)	178,822 (7.8)	13,801 (4.9)	5,561 (5.8)	14,694 (5.3)	1,294 (6.6)
CABG	197,848 (6.7)	166,180 (7.3)	9,830 (3.5)	5,295 (5.6)	15,296 (5.5)	1,247 (6.4)
Heart valve surgery	65,260 (2.2)	57,126 (2.5)	2,603 (0.9)	1,190 (1.3)	4,063 (1.5)	278 (1.4)
Left ventricular asist device	785 (0.03)	575 (0.03)	125 (0.04)	14 (0.01)	67 (0.02)	4 (0.02)
Kidney transplant	15,832 (0.5)	9,385 (0.4)	2,550 (0.9)	1,012 (1.1)	2,698 (1)	187 (1)

© 2025 Glance LG et al. *JAMA Network Open*.

Supplement Table 2. Patient Characteristics.						
	Total	White	Black	Asian	Hispanic	Native American/ Alaska Native
	(N=2,964,517)	(N=2,288,003)	(N=282,646)	(N=95,308)	(N=279,011)	(N=19,549)
**Characteristics**						
**Intensive care unit**						
None	1,710,434 (57.7)	1,321,485 (57.8)	160,985 (57)	57,233 (60.1)	159,630 (57.2)	11,101 (56.8)
Intermediate care	651,291 (22)	496,594 (21.7)	60,085 (21.3)	21,296 (22.3)	69,127 (24.8)	4,189 (21.4)
Intensive care	602,792 (20.3)	469,924 (20.5)	61,576 (21.8)	16,779 (17.6)	50,254 (18)	4,259 (21.8)
**In-hospital complilcations**						
Acute myocardial infarction	18,977 (0.6)	14,873 (0.7)	1,643 (0.6)	673 (0.7)	1,648 (0.6)	140 (0.7)
Congestive heart failure	49,438 (1.7)	41,957 (1.8)	3,224 (1.1)	1,214 (1.3)	2,759 (1)	284 (1.5)
Stroke	11,351 (0.4)	8,164 (0.4)	1,675 (0.6)	435 (0.5)	1,007 (0.4)	70 (0.4)
Acute renal failure	76,125 (2.6)	58,189 (2.5)	9,077 (3.2)	2,158 (2.3)	6,237 (2.2)	464 (2.4)
Acute respiratory failure	110,121 (3.7)	87,321 (3.8)	10,320 (3.7)	3,171 (3.3)	8,645 (3.1)	664 (3.4)
**Hospital characteristics**						
Hospital percentage of Black patients						
0-4.9%	1,197,674 (40.4)	1,006,167 (44)	15,965 (5.7)	39,058 (41)	122,849 (44)	13,635 (69.8)
5.0-9.9%	514,106 (17.3)	422,792 (18.5)	24,970 (8.8)	15,353 (16.1)	48,586 (17.4)	2,405 (12.3)
10.0-24.9%	778,360 (26.3)	587,123 (25.7)	86,384 (30.6)	29,794 (31.3)	72,353 (25.9)	2,706 (13.8)
25.0-49.9%	372,874 (12.6)	234,792 (10.3)	99,059 (35.1)	9,666 (10.1)	28,630 (10.3)	727 (3.7)
50.0% -	101,503 (3.4)	37,129 (1.6)	56,268 (19.9)	1,437 (1.5)	6,593 (2.4)	76 (0.4)
Disproportionate share percentage (DSH)						
0-9.9%	119,552 (4)	100,545 (4.4)	5,133 (1.8)	4,658 (4.9)	8,982 (3.2)	234 (1.2)
10.0-24.9%	838,742 (28.3)	711,408 (31.1)	56,700 (20.1)	20,946 (22)	46,659 (16.7)	3,029 (15.5)
25.0-49.9%	1,259,196 (42.5)	969,996 (42.4)	137,649 (48.7)	36,026 (37.8)	105,809 (37.9)	9,716 (49.7)
50.0%-	747,027 (25.2)	506,054 (22.1)	83,164 (29.4)	33,678 (35.3)	117,561 (42.1)	6,570 (33.6)
Resident-to-bed ratio						
0	1,105,204 (37.3)	884,885 (38.7)	83,166 (29.4)	30,009 (31.5)	98,457 (35.3)	8,687 (44.4)
>0-0.10	641,815 (21.7)	493,849 (21.6)	54,283 (19.2)	20,970 (22)	67,740 (24.3)	4,973 (25.4)
0.11-0.20	240,383 (8.1)	185,927 (8.1)	26,141 (9.3)	8,250 (8.7)	19,151 (6.9)	914 (4.7)
0.21-0.40	245,513 (8.3)	181,516 (7.9)	33,395 (11.8)	8,818 (9.3)	21,293 (7.6)	491 (2.5)
0.41-	731,602 (24.7)	541,826 (23.7)	85,661 (30.3)	27,261 (28.6)	72,370 (25.9)	4,484 (22.9)
Average daily census						
<=50	308,182 (10.4)	264,260 (11.6)	17,162 (6.1)	4,577 (4.8)	17,680 (6.3)	4,503 (23)
51-150	800,071 (27)	631,335 (27.6)	63,911 (22.6)	26,551 (27.9)	73,187 (26.2)	5,087 (26)
151-300	753,433 (25.4)	569,389 (24.9)	75,058 (26.6)	26,582 (27.9)	78,919 (28.3)	3,485 (17.8)
301-	1,102,831 (37.2)	823,019 (36)	126,515 (44.8)	37,598 (39.5)	109,225 (39.2)	6,474 (33.1)
Region						
New England	128,896 (4.4)	115,671 (5.1)	4,715 (1.7)	2,098 (2.2)	6,311 (2.3)	101 (0.5)
Middle Atlantic	374,453 (12.6)	293,856 (12.8)	36,714 (13)	12,728 (13.4)	30,729 (11)	426 (2.2)
South Atlantic	595,961 (20.1)	455,533 (19.9)	93,059 (32.9)	8,435 (8.9)	38,100 (13.7)	834 (4.3)
East North Central	379,073 (12.8)	325,807 (14.2)	36,492 (12.9)	4,695 (4.9)	11,338 (4.1)	741 (3.8)
East South Central	197,974 (6.7)	168,439 (7.4)	27,222 (9.6)	875 (0.9)	1,294 (0.5)	144 (0.7)
West North Central	227,552 (7.7)	205,084 (9)	12,791 (4.5)	2,861 (3)	4,560 (1.6)	2,256 (11.5)
West South Central	350,663 (11.8)	235,626 (10.3)	40,263 (14.3)	6,219 (6.5)	63,771 (22.9)	4,784 (24.5)
Mountain	212,709 (7.2)	169,885 (7.4)	5,994 (2.1)	4,109 (4.3)	26,633 (9.6)	6,088 (31.1)
Pacific	487,293 (16.4)	318,058 (13.9)	25,391 (9)	53,287 (55.9)	86,383 (31)	4,174 (21.4)
Puerto Rico	9,943 (0.3)	44 (0)	5 (0)	1 (0)	9,892 (3.55)	1 (0.01)
**Outcome**						
Long-term Nursing Home Stay or Death	269,541 (9.1)	211,333 (9.2)	31,020 (11)	7,327 (7.7)	18,472 (6.6)	1,389 (7.1)

© 2025 Glance LG et al. *JAMA Network Open.*

Supplement Table 3. Hospital Characteristics

Characteristics	Hospitals, No. (%)
Hospital percentage of Black patients	
0-4.9%	1,564 (48.7)
5.0-9.9%	441 (13.7)
10.0-24.9%	660 (20.6)
25.0-49.9%	379 (11.8)
50.0% -	165 (5.1)
Disproportionate share percentage (DSH)	
0-9.9%	219 (6.8)
10.0-24.9%	1,046 (32.6)
25.0-49.9%	1,542 (48.1)
50.0%-	402 (12.5)
Resident-to-bed ratio	
0	1,988 (62)
>0-0.10	558 (17.4)
0.11-0.20	207 (6.5)
0.21-0.40	228 (7.1)
0.41-	228 (7.1)
Average daily census	
<=50	1,318 (41.1)
51-150	1,026 (32)
151-300	538 (16.8)
301-	327 (10.2)
Region	
New England	131 (4.1)
Middle Atlantic	350 (10.9)
South Atlantic	567 (17.7)
East North Central	28 (0.9)
East South Central	290 (9)
West North Central	692 (21.6)
West South Central	482 (15)
Mountain	229 (7.1)
Pacific	391 (12.2)
Puerto Rico	49 (1.5)

© 2025 Glance LG et al. *JAMA Network Open*.

Supplement Table 4. Long-term Nursing Home Stay or Death

	Unadusted		Model 1		Model 2		Model 3	
	OR (95% CI)	P	OR (95% CI)	P	OR (95% CI)	P	OR (95% CI)	P
Race								
White	reference		reference		reference		reference	
Black	1.33 (1.3 ,1.37)	0.000	1.09 (1.06 ,1.12)	0.000	1.09 (1.06 ,1.12)	0.000	1.06 (1.04 ,1.08)	0.000
Asian/Pacific Islander	0.79 (0.75 ,0.83)	0.000	0.72 (0.68 ,0.76)	0.000	0.73 (0.69 ,0.77)	0.000	0.79 (0.74 ,0.83)	0.000
Hispanic	0.72 (0.7 ,0.74)	0.000	0.66 (0.64 ,0.68)	0.000	0.67 (0.65 ,0.69)	0.000	0.71 (0.7 ,0.73)	0.000
American Indian/Alsaka Native	0.79 (0.72 ,0.87)	0.000	0.74 (0.67 ,0.82)	0.000	0.75 (0.69 ,0.82)	0.000	0.82 (0.75 ,0.9)	0.000
Time (3 months)	0.96 (0.96 ,0.962)	0.000	0.96 (0.957 ,0.959)	0.000	0.96 (0.957 ,0.96)	0.000	0.96 (0.957 ,0.959)	0.000
Intercept shift during the pandemic (post)	0.98 (0.96 ,1.01)	0.258	0.97 (0.94 ,0.99)	0.021	0.96 (0.93 ,0.99)	0.013	0.97 (0.94 ,0.99)	0.022
Slope shift during the pandemic	1.02 (1.01 ,1.03)	0.000	1.02 (1.02 ,1.03)	0.000	1.03 (1.02 ,1.04)	0.000	1.03 (1.023 ,1.04)	0.000
Intercept shift by race and ethnicity								
Post. X White	reference		reference		reference		reference	
Post X Black	0.92 (0.86 ,0.99)	0.025	0.97 (0.9 ,1.05)	0.447	0.96 (0.89 ,1.04)	0.326	0.96 (0.89 ,1.04)	0.336
Post X Asian/Pacific Islander	1.05 (0.91 ,1.2)	0.517	1.04 (0.91 ,1.2)	0.536	1.05 (0.92 ,1.2)	0.490	1.04 (0.91 ,1.2)	0.55
Post XHispanic	1.05 (0.96 ,1.16)	0.262	1.09 (0.99 ,1.2)	0.075	1.082 (0.98 ,1.19)	0.102	1.08 (0.98 ,1.19)	0.113
Post X American Indian/Alsaka Native	1.07 (0.77 ,1.48)	0.692	1.05 (0.76 ,1.44)	0.786	1.03 (0.75 ,1.43)	0.844	1.04 (0.75 ,1.43)	0.818
Slope shift by race and ethnicity								
Slope X White	reference		reference		reference		reference	
Slope shift X Black	1.03 (1.01 ,1.05)	0.010	1.02 (1 ,1.04)	0.100	1.02 (1 ,1.04)	0.065	1.02 (1 ,1.04)	0.062
Slope shift X Asian/Pacific Islander	1 (0.96 ,1.04)	0.910	1 (0.96 ,1.04)	0.813	0.99 (0.95 ,1.03)	0.732	0.99 (0.95 ,1.03)	0.598
Slope shift X Hispanic	1.01 (0.98 ,1.04)	0.603	1 (0.98 ,1.03)	0.780	1.01 (0.98 ,1.03)	0.689	1 (0.97 ,1.03)	0.867
slope shift X American Indian/Alsaka Native	1.05 (0.96 ,1.15)	0.320	1.05 (0.96 ,1.15)	0.269	1.05 (0.96 ,1.15)	0.259	1.05 (0.96 ,1.14)	0.31
Age								
65-69	reference		reference		reference		reference	
70-74	1.06 (1.04 ,1.07)	0.000	1.12 (1.1 ,1.13)	0.000	1.12 (1.1 ,1.14)	0.000	1.12 (1.11 ,1.14)	0.000
75-79	1.29 (1.27 ,1.3)	0.000	1.33 (1.31 ,1.35)	0.000	1.35 (1.33 ,1.37)	0.000	1.35 (1.33 ,1.37)	0.000
80-84	1.64 (1.61 ,1.66)	0.000	1.65 (1.62 ,1.68)	0.000	1.68 (1.66 ,1.71)	0.000	1.69 (1.66 ,1.71)	0.000
85-89	2.11 (2.08 ,2.15)	0.000	2.04 (2 ,2.07)	0.000	2.1 (2.06 ,2.14)	0.000	2.1 (2.06 ,2.13)	0.000
90-94	2.51 (2.46 ,2.56)	0.000	2.34 (2.29 ,2.39)	0.000	2.45 (2.4 ,2.51)	0.000	2.45 (2.39 ,2.5)	0.000
95-	2.75 (2.67 ,2.82)	0.000	2.47 (2.4 ,2.55)	0.000	2.63 (2.55 ,2.71)	0.000	2.62 (2.54 ,2.7)	0.000
Female	1.11 (1.1 ,1.12)	0.000	1.1 (1.09 ,1.11)	0.000	1.1 (1.09 ,1.11)	0.000	1.1 (1.09 ,1.11)	0.000

© 2025 Glance LG et al. *JAMA Network Open*.

Supplement Table 4. Long-term Nursing Home Stay or Death								
	Unadusted		Model 1		Model 2		Model 3	
	OR (95% CI)	P	OR (95% CI)	P	OR (95% CI)	P	OR (95% CI)	P
Functional Status/frailty								
Wheelchair			1.24 (1.19 ,1.3)	0.000	1.27 (1.22 ,1.33)	0.000	1.29 (1.24 ,1.35)	0.000
Oxygen supplementation			0.89 (0.87 ,0.91)	0.000	0.9 (0.88 ,0.92)	0.000	0.9 (0.88 ,0.92)	0.000
Malnutrition			1.68 (1.61 ,1.75)	0.000	1.46 (1.41 ,1.51)	0.000	1.47 (1.42 ,1.52)	0.000
Senility			1.23 (1.15 ,1.31)	0.000	1.23 (1.15 ,1.31)	0.000	1.23 (1.15 ,1.31)	0.000
Urinary incontinence			1.23 (1.2 ,1.27)	0.000	1.26 (1.23 ,1.3)	0.000	1.27 (1.23 ,1.3)	0.000
Fecal incontinence			1.52 (1.43 ,1.61)	0.000	1.51 (1.42 ,1.6)	0.000	1.51 (1.43 ,1.61)	0.000
Gait disturbane			1.55 (1.47 ,1.62)	0.000	1.59 (1.51 ,1.67)	0.000	1.53 (1.45 ,1.61)	0.000
Cognitive dysfunction			1.39 (1.36 ,1.43)	0.000	1.38 (1.34 ,1.41)	0.000	1.38 (1.35 ,1.42)	0.000
Dependent on provider			1 (0.96 ,1.05)	0.000	1.02 (0.98 ,1.07)	0.268	1.05 (1.01 ,1.1)	0.007
Housing instability			2.69 (2.48 ,2.92)	0.854	2.81 (2.59 ,3.05)	0.000	2.97 (2.74 ,3.21)	0.000
Social environment			1.75 (1.64 ,1.87)	0.000	1.8 (1.69 ,1.92)	0.000	1.81 (1.69 ,1.93)	0.000
Pre-existing Conditions								
Congestive heart failure								
None			reference		reference		reference	
Systolic CHF			1.33 (1.3 ,1.35)	0.000	1.29 (1.26 ,1.31)	0.000	1.28 (1.26 ,1.3)	0.000
Diastolic CHF			1.23 (1.21 ,1.25)	0.000	1.22 (1.2 ,1.24)	0.000	1.21 (1.19 ,1.22)	0.000
Systolic and diastolic CHF			1.31 (1.27 ,1.34)	0.000	1.27 (1.23 ,1.3)	0.000	1.27 (1.24 ,1.3)	0.000
Unspecified CHF			1.17 (1.14 ,1.19)	0.000	1.16 (1.14 ,1.18)	0.000	1.16 (1.14 ,1.18)	0.000
Myocardial infarction								
No prior myocardial infarction			reference		reference		reference	
Prior ST-segment elevation MI			1.35 (1.22 ,1.48)	0.000	1.17 (1.07 ,1.29)	0.001	1.17 (1.06 ,1.29)	0.001
Prior Non-ST-segment elevation MI			1.31 (1.28 ,1.34)	0.000	1.2 (1.17 ,1.23)	0.000	1.19 (1.16 ,1.22)	0.000
Prior other MI			1.27 (1.23 ,1.31)	0.000	1.19 (1.15 ,1.22)	0.000	1.17 (1.14 ,1.21)	0.000
Pulmonary								
Acute respiratory distress syndrome			1.1 (1.02 ,1.19)	0.015	0.92 (0.85 ,0.99)	0.036	0.94 (0.87 ,1.01)	0.088
Pulmonary edema			1.07 (1.03 ,1.12)	0.000	1 (0.96 ,1.04)	0.908	1.02 (0.98 ,1.06)	0.33
Pulmonary interstitial disease			1.01 (0.98 ,1.04)	0.568	1 (0.97 ,1.03)	0.864	1.01 (0.98 ,1.04)	0.693
Acute respiratory failure			1.03 (1.02 ,1.04)	0.000	0.96 (0.95 ,0.98)	0.000	0.97 (0.96 ,0.98)	0.000
Acute on chronic respiratory failure			1.12 (1.1 ,1.14)	0.000	1.05 (1.03 ,1.07)	0.000	1.06 (1.04 ,1.08)	0.000
Chronic respiratory failure			1.01 (0.98 ,1.05)	0.422	1.04 (1.01 ,1.08)	0.011	1.06 (1.02 ,1.09)	0.001
Respiratory failure, unspecified			1.14 (1.09 ,1.19)	0.000	1.08 (1.03 ,1.13)	0.001	1.08 (1.03 ,1.13)	0.001

© 2025 Glance LG et al. *JAMA Network Open*.

Supplement Table 4. Long-term Nursing Home Stay or Death

	Unadusted		Model 1		Model 2		Model 3	
	OR (95% CI)	P	OR (95% CI)	P	OR (95% CI)	P	OR (95% CI)	P
COVID-19			0.89 (0.86 ,0.92)	0.000	0.9 (0.87 ,0.93)	0.000	0.88 (0.85 ,0.91)	0.000
Elixhauser comorbidities								
Acquired immune deficiency syndrome			1.13 (1.03 ,1.23)	0.007	1.14 (1.05 ,1.24)	0.003	1.1 (1.01 ,1.2)	0.023
Alcohol abuse			1.45 (1.42 ,1.49)	0.000	1.39 (1.36 ,1.43)	0.000	1.41 (1.37 ,1.44)	0.000
Anemia, nutritional deficiency			1.19 (1.18 ,1.2)	0.000	1.18 (1.17 ,1.19)	0.000	1.18 (1.17 ,1.19)	0.000
Anemia, iron deficiency			1.22 (1.17 ,1.28)	0.000	1.2 (1.15 ,1.25)	0.000	1.19 (1.14 ,1.24)	0.000
Lymphoma			0.97 (0.94 ,1)	0.052	0.98 (0.95 ,1.01)	0.158	0.98 (0.95 ,1.01)	0.224
Leukemia			1.01 (0.97 ,1.04)	0.632	1.01 (0.98 ,1.05)	0.457	1.01 (0.98 ,1.05)	0.412
Metastatic cancer			1.46 (1.43 ,1.49)	0.000	1.48 (1.44 ,1.51)	0.000	1.48 (1.45 ,1.51)	0.000
Solid tumor, in situ			0.98 (0.78 ,1.23)	0.874	1.01 (0.81 ,1.27)	0.914	1.02 (0.82 ,1.28)	0.835
Solid tumor, without metstasis			1.12 (1.1 ,1.14)	0.000	1.13 (1.11 ,1.15)	0.000	1.13 (1.11 ,1.15)	0.000
Cerebrovascular disease			1.19 (1.16 ,1.22)	0.000	1.19 (1.16 ,1.21)	0.000	1.2 (1.18 ,1.23)	0.000
Coagulopathy			1.09 (1.07 ,1.1)	0.000	1.05 (1.03 ,1.06)	0.000	1.05 (1.04 ,1.06)	0.000
Dementia			1.65 (1.63 ,1.68)	0.000	1.69 (1.67 ,1.72)	0.000	1.7 (1.67 ,1.72)	0.000
Depression			1.09 (1.08 ,1.11)	0.000	1.11 (1.09 ,1.12)	0.000	1.11 (1.09 ,1.12)	0.000
Diabetes with chronic complications			1.27 (1.25 ,1.28)	0.000	1.26 (1.24 ,1.27)	0.000	1.26 (1.25 ,1.28)	0.000
Liver disease, mild			1 (0.98 ,1.02)	0.741	1 (0.98 ,1.02)	0.915	1.01 (0.99 ,1.03)	0.465
Liver disease and failure, moderate to severe			1.29 (1.25 ,1.34)	0.000	1.27 (1.22 ,1.31)	0.000	1.28 (1.23 ,1.32)	0.000
Neurologic disorder affecting movement			1.21 (1.18 ,1.23)	0.000	1.21 (1.19 ,1.24)	0.000	1.22 (1.2 ,1.24)	0.000
Neurologic disorder, other			1.63 (1.61 ,1.65)	0.000	1.57 (1.55 ,1.59)	0.000	1.58 (1.56 ,1.59)	0.000
Seizures and epilepsy			1.13 (1.1 ,1.15)	0.000	1.11 (1.08 ,1.13)	0.000	1.1 (1.07 ,1.12)	0.000
Obesity			1.22 (1.2 ,1.23)	0.000	1.2 (1.19 ,1.22)	0.000	1.2 (1.19 ,1.22)	0.000
Paralysis			1.35 (1.32 ,1.38)	0.000	1.33 (1.3 ,1.37)	0.000	1.32 (1.28 ,1.35)	0.000
Peripheral vascular disease			1.05 (1 ,1.1)	0.042	1.07 (1.03 ,1.11)	0.001	1.11 (1.08 ,1.14)	0.000
Psychoses			1.48 (1.43 ,1.54)	0.000	1.52 (1.47 ,1.57)	0.000	1.54 (1.5 ,1.59)	0.000
Renal failure, moderate			1.04 (1.03 ,1.05)	0.000	1.04 (1.03 ,1.05)	0.000	1.04 (1.03 ,1.05)	0.000
Renal failure, severe			1.34 (1.31 ,1.36)	0.000	1.32 (1.3 ,1.34)	0.000	1.32 (1.3 ,1.34)	0.000
Peptic ulcer disease with bleeding			1.47 (1.42 ,1.51)	0.000	1.34 (1.3 ,1.38)	0.000	1.34 (1.3 ,1.38)	0.000
Valvular disease			1.12 (1.1 ,1.13)	0.000	1.08 (1.06 ,1.1)	0.000	1.07 (1.06 ,1.09)	0.000
Weight loss			1.1 (1.06 ,1.15)	0.000	1.21 (1.17 ,1.25)	0.000	1.2 (1.17 ,1.24)	0.000

© 2025 Glance LG et al. *JAMA Network Open*.

Supplement Table 4. Long-term Nursing Home Stay or Death								
	Unadusted		Model 1		Model 2		Model 3	
	OR (95% CI)	P	OR (95% CI)	P	OR (95% CI)	P	OR (95% CI)	P
Prior procedures								
PCI			0.78 (0.76 ,0.79)	0.000	0.79 (0.78 ,0.8)	0.000	0.78 (0.77 ,0.79)	0.000
CABG			0.87 (0.85 ,0.89)	0.000	0.88 (0.86 ,0.9)	0.000	0.87 (0.86 ,0.89)	0.000
Heart valve surgery			0.86 (0.83 ,0.89)	0.000	0.89 (0.87 ,0.92)	0.000	0.89 (0.86 ,0.92)	0.000
Left ventricular asist device			0.69 (0.5 ,0.95)	0.024	0.67 (0.48 ,0.92)	0.013	0.69 (0.5 ,0.94)	0.020
Kidney transplant			0.69 (0.65 ,0.74)	0.000	0.71 (0.67 ,0.76)	0.000	0.72 (0.67 ,0.77)	0.000
Intensive care unit								
None					reference		reference	
Intermediate care					1.25 (1.23 ,1.28)	0.000	1.28 (1.26 ,1.3)	0.000
Intensive care					1.53 (1.5 ,1.56)	0.000	1.51 (1.49 ,1.53)	0.000
In-hospital complilcations								
Acute myocardial infarction					1.16 (1.11 ,1.21)	0.000	1.15 (1.1 ,1.21)	0.000
Congestive heart failure					1.43 (1.39 ,1.47)	0.000	1.4 (1.36 ,1.43)	0.000
Stroke					1.99 (1.9 ,2.09)	0.000	2 (1.9 ,2.1)	0.000
Acute renal failure					1.31 (1.28 ,1.35)	0.000	1.31 (1.28 ,1.34)	0.000
Acute respiratory failure					1.35 (1.32 ,1.38)	0.000	1.35 (1.33 ,1.38)	0.000
**Hospital characteristics**								
Hospital percentage of Black patients								
0-4.9%							reference	
5.0-9.9%							1.05 (1.02 ,1.08)	0.004
10.0-24.9%							1.05 (1.02 ,1.08)	0.003
25.0-49.9%							1.02 (0.98 ,1.06)	0.272
50.0% -							1.07 (1.01 ,1.12)	0.019
Disproportionate share percentage (DSH)								
0-9.9%							reference	
10.0-24.9%							1.16 (1.1 ,1.22)	0.000
25.0-49.9%							1.24 (1.18 ,1.31)	0.000
50.0%-							1.38 (1.3 ,1.46)	0.000

© 2025 Glance LG et al. *JAMA Network Open*.

Supplement Table 4. Long-term Nursing Home Stay or Death								
	Unadusted		Model 1		Model 2		Model 3	
	OR (95% CI)	P	OR (95% CI)	P	OR (95% CI)	P	OR (95% CI)	P
Resident-to-bed ratio								
0							reference	
>0-0.10							0.98 (0.95 ,1)	0.083
0.11-0.20							1.03 (0.99 ,1.07)	0.196
0.21-0.40							0.96 (0.92 ,1)	0.072
0.41-							0.94 (0.9 ,0.98)	0.006
Average daily census								
<=50							reference	
51-150							0.97 (0.94 ,1)	0.031
151-300							0.96 (0.93 ,0.99)	0.023
301-							0.89 (0.85 ,0.92)	0.000
Region								
New England							reference	
Middle Atlantic							1.13 (1.08 ,1.19)	0.000
South Atlantic							0.85 (0.81 ,0.89)	0.000
East North Central							0.98 (0.94 ,1.03)	0.527
East South Central							0.85 (0.8 ,0.9)	0.000
West North Central							0.91 (0.86 ,0.95)	0.000
West South Central							0.79 (0.75 ,0.83)	0.000
Mountain							0.72 (0.68 ,0.76)	0.000
Pacific							0.72 (0.68 ,0.76)	0.000
Puerto Rico							0.17 (0.14 ,0.2)	0.000

© 2025 Glance LG et al. *JAMA Network Open*.
