# Supplementary material for: COVID-19 Pandemic and Racial and Ethnic Disparities in Long-Term Nursing Home Stay or Death Following Hospital Discharge
Source: JAMA Netw Open. 2025 Jan 24;8(1):e2456816. doi: 10.1001/jamanetworkopen.2024.56816 (PMC11762228; doi:10.1001/jamanetworkopen.2024.56816)
Supplement: Supplement 2. — Data Sharing Statement [file jamanetwopen-e2456816-s002.pdf]

Data Sharing Statement

Glance. COVID-19 Pandemic and Racial and Ethnic Disparities in Long-Term Nursing Home Stay or Death Following Hospital Discharge. *JAMA Netw Open*. Published January 24, 2025. doi:10.1001/jamanetworkopen.2024.56816

Data
Data available: No

Additional Information
Explanation for why data not available: This data is from CMS and cannot be shared with others without a DUA.
